# Regioselective Photooxidation of Citronellol: A Way to Monomers for Functionalized Bio-Polyesters

**DOI:** 10.3389/fchem.2020.00085

**Published:** 2020-02-13

**Authors:** Deianira Lanteri, Silvia Quattrosoldi, Michelina Soccio, Andrea Basso, Dario Cavallo, Andrea Munari, Renata Riva, Nadia Lotti, Lisa Moni

**Affiliations:** ^1^Department of Chemistry and Industrial Chemistry, University of Genova, Genova, Italy; ^2^Department of Civil, Chemical, Environmental and Materials Engineering, University of Bologna, Bologna, Italy; ^3^Department of Pharmacy, University of Genova, Genova, Italy

**Keywords:** photooxidation, zeolites, terpenoids, poly(butylene succinate), copolymerization, renewable polymers

## Abstract

Dye-sensitized photooxygenation reaction of bio-based double bond-containing substrates is proposed as sustainable functionalization of terpenes and terpenoids to transform them into polyoxygenated compounds to be employed for the synthesis of new bio-based polyesters. As proof of concept, citronellol **1** has been regioselectively converted into diol **4** using singlet oxygen (^1^O_2_), a traceless reagent that can be generated from air, visible light and zeolite supported-photosensitizer (Thionine-NaY). With our synthetic approach, diol **4** has been obtained in two-steps, with good regioselectivity, using green reagents such as light and air, and finally a solvent-free oxidation step. From this compound, a citronellol-based copolyester of poly(butylene succinate) (PBS) has been synthesized and fully characterized. The results obtained evidence that the proposed copolymerization of PBS with the citronellol-based building blocks allows to obtain a more flexible and functionalizable material, by exploiting a largely available natural molecule modified through a green synthetic path.

## Introduction

Modern society is recording an urgent demand for sustainable, simple and selective synthetic methodologies for the preparation of smart materials and added value compounds (Corma et al., 2007; Kohli et al., 2019).

In the polymer field, the interest for fully bio-based materials has enormously grown up, due to the urgent need to solve the serious environmental pollution problems caused by the extensive use of traditional fossil-based plastics. Poly(butylene succinate) (PBS), synthesized through the well-known two-stage melt polycondensation reaction from 1,4-butanediol and succinic acid, has attracted much attention as promising ecofriendly bioplastic, being characterized by intriguing physical and mechanical properties, similar to those of polyethylene (LDPE), good processability, low cost, and biodegradability. Indeed, PBS has been demonstrated to be suitable for a wide range of different applications, ranging from biomedicine to packaging. It is also interesting to note that bio 1,4-butanediol can be obtained from bio-based succinic acid, this latter derived from biomass or sugar substrates.

In recent years, many works have been published describing the use of PBS in regenerative medicine and for the realization of nanosystems for controlled drug release (Gigli et al., 2016). In this context the biocompatibility of the material is crucial, but also the straightforward manipulation to bind biological molecules is of great importance. This property can be achieved through insertion of a comonomer unit in the PBS polymer chain that permits post functionalization. Copolymerization has always been a viable tool (Gigli et al., 2012; Gualandi et al., 2012; Soccio et al., 2012) in this view. It is worth to notice that by means of this strategy, at the same time, it is possible to reduce the stiffness of the final material, through a decrease in the degree of crystallinity.

**Scheme 1:**
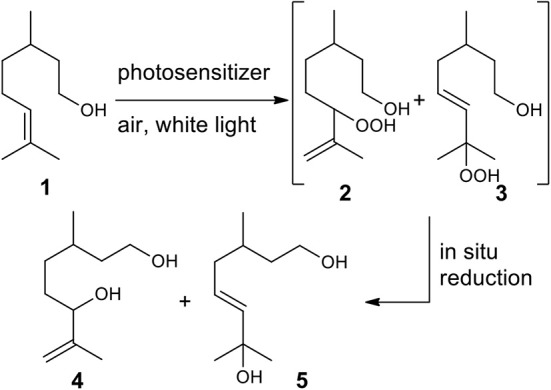
Photooxygenation reaction of (rac-)citronellol **1** and subsequent reduction of hydroperxides **2** and **3** to give diols **4** and **5**.

Within the hydrocarbon-rich biomass, terpenes, and terpenoids represent a vast class of molecules, which are finding wide application in polymer science. These compounds are considered interesting organic feedstock for the generation of green plastics and composites (Wilbon et al., 2013; Winnacker and Rieger, 2015), due to their natural abundance and low-cost. Moreover, new approaches, such as the controlled and sustainable functionalization, have been recently proposed to transform them into monomers for alternative polymerization process, to produce even more competitive polymers (Thomsett et al., 2016). Among all the strategies reported in the literature (Byrne et al., 2004; Zhang et al., 2005; Kobayashi et al., 2009; Lowe, 2010; Firdaus et al., 2011; Firdaus and Meier, 2013; Hauenstein et al., 2016; Roth et al., 2017; Parrino et al., 2018; Stamm et al., 2019; Thomsett et al., 2019), to the best of our knowledge the dye-sensitized photooxygenation of terpenes and terpenoids to obtain bio-based polyhydroxylated monomers and their use for the production of functionalized polyesters, has never been reported. Reactions of singlet oxygen with C-C double bounds are well-known and documented in literature (Schenck et al., 1953; Foote and Wexler, 1964; Alberti and Orfanopoulos, 2010). Since singlet oxygen (^1^O_2_) can be conveniently generated by irradiation of oxygen naturally present in the atmosphere, we envisioned to obtain diols, simply by treatment of terpenes and terpenoids with air, light and a photosensitizer, followed by reduction of the resulting alcohols. Here we present a proof of concept of our idea, describing the photosensitized conversion of (*rac*-)citronellol **1** into diol **4**, this latter employed to prepare a PBS-based copolymer. The photooxidation of citronellol **1** using ^1^O_2_ has been well-studied since 90's (Bouamri et al., 1991), as it represents one of the few photochemical processes used at industrial scale, to produce rose oxide, an important fragrance (Monnerie and Ortner, 2001). Typically, the bulk synthesis is conducted in batch, employing Rose Bengal (RB) as sensitizer, an alcoholic solvent, and white lamp or sunlight as light sources. Due to the instability of the hydroxyperoxides **2** and **3**, the corresponding alcohols **4** and **5** are isolated after *in situ* reduction of the reaction mixture (Scheme 1). Recently, the process has been implemented thanks to the use flow methods (Maurya et al., 2011; Park et al., 2015; Clark et al., 2016; Ioannou et al., 2017; Anselmo et al., 2019), achieving increased efficiency compared to the batch conditions. However, the potential synthetic use of this reaction is suppressed by its poor selectivity, whereas in all cases a mixture of secondary and tertiary alcohols **4** and **5** is obtained in almost equimolar ratio (Maurya et al., 2011; Park et al., 2015; Clark et al., 2016; Ioannou et al., 2017). Moreover, the ^1^O_2_ photooxidation of **1** suffers safety problems when pure oxygen must be employed, affecting the scalability of the process.

Regioselectivity in photooxygenation reactions has been successfully achieved for simple trisubstituted alkenes by performing them in a confined space, using molecular containers such as self-assembled molecular hosts (Natarajan et al., 2007; Geer et al., 2013), zeolites (Shailaja et al., 2000; Stratakis and Froudakis, 2000; Kaanumalle et al., 2002; Chen et al., 2005), or microemulsions (Nardello et al., 2004). The confined space allows to better control the conformation of alkene and thus the accessibility of the allylic hydrogen(s) with respect to the attacking singlet oxygen during the hydroperoxide formation. Anyway, in most of these studies, only simple model alkenes have been used in analytical amount, and no scalability of the process has been investigated.

Herein, we describe the photooxidation of **1** using air, visible light and zeolite supported-photosensitizer (Thionine-NaY). With our synthetic approach, diol **4** has been obtained in two-steps, with good regioselectivity, using green reagents, such as light and air, and a solvent-free oxidation step. Moreover, the supported catalyst can be recovered by filtration and reused without meaningful loss of activity.

Using diol **4**, a random copolyester was synthetized by a solvent free two-stage polycondensation process, starting from a mixture of **4/**1,4-butandiol (BD), and dimethyl succinate (DMS). Furthermore, we demonstrated the possibility to post-functionalize the new citronellol-based copolyester with different thiols applying the UV-mediated thiol-ene click reaction (Dondoni, 2008; Hoyle and Bowman, 2010). Since the introduction of additional functional groups into bio-polyesters represents an important tool to increase their potential for biomedical applications and, at the same time, is still a challenge (Parrish and Emrick, 2006; Williams, 2007; Jérôme and Lecomte, 2008; Pounder and Dove, 2010), our approach can be considered a new method to obtain easily functionalizable copolyesters.

## Materials and Methods

### General Remarks

NMR spectra were taken at RT in CDCl_3_ at 300 MHz (^1^H), and 75 (^13^C), using, as internal standard, TMS (^1^H NMR: 0.000 ppm) or the central peak of CDCl_3_ (^13^C: 77.02 ppm). Chemical shifts are reported in ppm (δ scale). Peak assignments were made with the aid of gCOSY and gHSQC experiments. GC–MS analyses were carried out on a Hewlett Packard 5890 Series II, using a HP-1 column, coupled with a HP-5971A spectrometer (electron impact). Analysis conditions are as follows: flow (He) 0.9 mL/min; initial temperature 70°C; initial time 2 min; gradient temperature 20°C /min; final temperature 260°C; final time 5 min. Photooxidation reactions were performed with a white lamp (300 Osram ultra-vitalux 230 V AC). Photoinduced thiol-ene reactions were performed with a Southern New England Ultraviolet Company Rayonet® apparatus equipped with 8 Iles Optical lamps (254 nm). TLC analyses were carried out on silica gel plates and viewed at UV (254 nm) and developed with Hanessian stain (dipping into a solution of (NH_4_)_4_MoO_4_.4 H_2_O (21 g) and Ce(SO_4_)_2_.4 H_2_O (1 g) in H_2_SO_4_ (31 ml) and H_2_O (469 ml) and warming) or with KMnO_4_. Rf were measured after an elution of 7–9 cm. Column chromatographies were done with the “flash” methodology using 220–400 mesh silica (Still et al., 1978). Petroleum ether (40–60°C) is abbreviated as PE. Citronellol, thionin acetate, methylene blue and zeolite Y, sodium (NaY) were purchased from Alfa Aesar; triphenylphosphine, triphenylphosphine polymer-bound, NaBH_4_ and 2,2-dimethoxy-2-phenylacetophenone were purchased from Sigma Aldrich; α-thioglycerol and cysteamine were purchased from TCI. All reagents are used as received.

Intrinsic viscosity was determined by using Ubbelohde type viscometer 31 13/Ic (diameter 0.84 mm) at 30°C. Four different polymer/CHCl_3_ solutions were tested (0.80, 0.69, 0.58, and 0.47 g/dl).

TGA analysis was carried out with a Perkin Elmer TGA7 Instrument under N_2_ flow (40 ml/min) by heating from 40 to 800°C at 10°C /min.

DSC measurements were conducted with a Perkin Elmer DSC7 Instrument. The external block temperature control was set at −120°C. The samples, about 10 mg, were encapsulated in aluminum pans and subjected to the following thermal treatment: i) heating from −70°C to a temperature 40°C above the melting, 20°C /min; ii) cooling to −70°C, 100°C /min; iii) heating from −70°C to a temperature 40°C above the melting, 20°C /min.

Wide angle X-ray scattering (WAXS) measurements were carried out with a PANalytical X'PertPro diffractometer equipped with a fast solid state X'Celerator detector and a copper target (λ = 0.15418 nm).

Stress-strain measurements were performed using an Instron 5966 tensile testing machine equipped with a 500 N load cell controlled by computer. Rectangular films (5 × 20 mm^2^) were employed and 10 mm/min crosshead speed was adopted.

Fourier-transform InfraRed (FTIR) spectra of PBS, copolymers and functionalized copolymer films were collected at RT, by means of a Bruker IFS66 spectrometer equipped with an attenuated total reflectance accessory (ATR). A total of 32 spectra in the spectral range 500–4,000 cm^−1^ with a resolution of 4 cm^−1^ were acquired for each sample.

### Photooxidation of Citronellol in Solution

A solution of **1** (500 mg, 3.20 mmol) and Rose Bengal (32 mg, 0.032 mmol) in MeOH (8 mL) was purged with air (dried on silica rods) and irradiated with white light. After consumption of the starting material (8 h, monitoring by TLC analysis), the mixture was cooled at 0°C, and NaBH_4_ (157 mg, 4.16 mmol) was added. After complete reduction of the hydroperoxides **2** and **3** (5 h, monitoring by TLC analysis), the reaction mixture was poured into 5% aqueous (NH_4_)H_2_PO_4_ and 1 M HCl (5:1, 15 mL), MeOH was evaporated, and the aqueous phases extracted twice with ethyl acetate. The organic phases were dried with Na_2_SO_4_, filtered and evaporated. The regioisomeric ratio was determined by ^1^H NMR of the crude product (**4**:**5** = 49:51). The crude was purified by column chromatography (PE/AcOEt from 3:2 to 1:1) affording **4** and **5** as a colorless oil (394 mg, 72%). Diol **4** (2 diast.): ^1^H NMR: δ_H_ 4.97–4.90 (2H, m, 1H of CH_2_=), 4.88–4.81 (2H, m, 1H of CH_2_=), 4.05 (2H, td, ^3^*J* = 6.4, 3.4 Hz, CH), 3.77–3.39 (4H, m, CH_2_), 1.73 (6H, s, CH_3_), 1.67–1.05 (18H, m, 3 CH_2_, 2 OH, CH), 0.92 (6H, d, ^3^*J* = 6.6 Hz, CH_3_); ^13^C NMR: δ_C_ 147.6 (C quat), 147.5 (C quat), 111.1 (CH_2_), 110.9 (CH_2_), 76.3 (CH), 75.9 (CH), 60.9 (2 CH_2_), 39.7 (2 CH_2_), 32.7 (CH_2_), 32.5 (CH_2_), 32.2 (CH_2_), 32.1 (CH_2_), 29.5 (CH), 29.2 (CH), 19.6 (2 CH_3_), 17.6 (CH_3_), 17.4 (CH_3_); GC-MS: 6.04 min, m/z (%): 154 (0.69) [M^+^-H_2_O], 99 (6.9), 86 (16), 84 (11), 83 (10), 72 (20), 71 (100), 70 (8.7), 69 (31), 68 (8.0), 67 (9.2), 58 (12), 57 (12), 56 (27), 55 (31), 43 (40), 42 (8.1), 41 (36), 39 (11); Diol **5**: ^1^H NMR: δ_H_ 5.67 – 5.54 (2H, m, 2 CH=), 3.80 – 3.60 (2H, m, CH_2_), 2.05 (1H, dt, ^3^*J* = 5.7, 4.2 Hz, 1H of CH_2_), 1.90 (1H, ddd, ^3^*J* = 9.5, 6.5, 4.3 Hz, 1H of CH_2_), 1.75 – 1.57 (4H, m, 2 OH, CH_2_), 1.42 (1H, dd, ^3^*J* = 11.1, 4.2 Hz, CH), 1.31 (6H, s, 2 CH_3_), 0.91 (3H, t, ^3^*J* = 4.7 Hz, CH_3_); ^13^C NMR: δ_C_ 139.6 (CH), 125.2 (CH), 70.6 (C quat), 60.8 (CH_2_), 39.6 (CH_2_), 39.2 (CH_2_), 29.8 (CH), 29.76 (CH_3_), 29.68 (CH_3_), 19.6 (CH_3_); GC-MS: 5.58 min, m/z (%): 172 (0.02) [M^+^], 139 (39), 121 (11), 109 (9.5), 95 (8.6), 93 (5.3), 85 (17), 84 (6.4), 83 (8.9), 82 (5.5), 81 (14), 71 (19), 69 (29), 67 (16), 59 (13), 57 (7.2), 55 (23), 53 (5.2), 43 (100), 41 (21), 38.9 (8.9).

### Loading Dye Within Zeolite

Supported photosensitizers on zeolite were prepared following a literature procedure (Shailaja et al., 2000): to a solution of the opportune photosensitizer (5 or 25 mg) in deionized water (250 mL), NaY (5.00 gr) is added. The mixture is mixed trough an orbital shaker for 24 h at room temperature in the dark. Then, the mixture is filtered, washed with deionized water and dried overnight at 110°C. During the cation exchange procedure, we assume the complete adsorption of the dye into the zeolite, although the obvious loss of organic molecule during washes can reduce the amount of the dye included into the final material.

### Photooxidation of Citronellol Using Thionine/NaY Photosensitizer

To a solution of **1** (100 mg, 0.64 mmol) in hexane (9 mL) TH/NaY (1.8 gr, 0.005% w/w) was added and the mixture was stirred in the dark for 15 min. Then the mixture was evaporated and dried using high vacuum pump for 30 min in the dark. The solid obtained was transferred on the suitable flask and connected to reactor (see Supplementary Material for details). The rotating mixture was purged with air (dried on silica rods) and irradiated with white light. After consumption of the starting material (19 h, monitoring by TLC analysis), the solid material was suspended in acetonitrile (9 mL) and stirred for 15 min in the dark. The mixture was then filtrated through a sintered funnel, and the liquid was treated with Ph_3_P (251 mg, 0.96 mmol). After complete reduction of the hydroperoxides **2** and **3** (1 h, monitoring by TLC analysis), the reaction mixture was evaporated, and the crude was purified by column chromatography (PE/AcOEt 1:1) affording **4** and **5** as a colorless oil (102 mg, 93%). The regioisomeric ratio was determined by ^1^H NMR of the crude product (**4**:**5** = 82:18).

### Recycling Tests of TH/NaY

To a solution of **1** (480 mg, 3.07 mmol) in hexane (44 mL) TH/NaY (8.7 gr, 0.005% w/w) was added and the mixture was stirred in the dark for 15 min. Then the mixture was evaporated and dried using high vacuum pump for 30 min in the dark. The solid obtained was transferred on the suitable flask and connected to reactor (see Supplementary Material for details). The rotating mixture was purged with air (dried on silica rods) and irradiated with white light. After consumption of the starting material (24 h, monitoring by TLC analysis), the solid material was suspended in acetonitrile (50 mL) and stirred for 15 min in the dark. The mixture was then filtrated through a sintered funnel and the liquid was treated with supported Ph_3_P (1.37 gr, 4.11 mmol, loading 3 mmol/gr). After complete reduction of the hydroperoxides **2** and **3** (3 days, monitoring by TLC analysis), the reaction mixture was filtrated on celite washing with CH_3_CN (5 mL), affording **4** and **5** as a colorless oil (416 mg, 78%). The regioisomeric ratio was determined by ^1^H NMR of the crude product (**4**:**5** = 89:11). The catalyst TH/NaY was dried at 110°C overnight and used for other two times with the following results: second run conversion 100%, ratio **4**:**5** = 77:23; third run conversion 100%, ratio **4**:**5** = 91:9.

### Synthesis of Copolyester 6

Diol **4** (850 mg, 4.93 mmol), butanediol (BD) (667 mg, 7.40 mmol), dimethyl succinate (DMS) (1.38 g, 9.49 mmol), together with Titanium(IV) butoxide (TBT) (9.50 mg, 0.078 mmol) were added in a stirred glass reactor with a thermostatted salt bath. In the first stage, the temperature was raised to 180°C, keeping the system under N_2_ atmosphere for 3 h. In the second stage, the temperature was slowly increased to 220°C, and the pressure was gradually reduced to 0.1 mbar. After 3 h, the reaction product was discharged from the reactor and cooled down to room temperature. A light yellow solid was obtained with 89% yield (1.70 g, 8.51 mmol).

### Synthesis of Copolyester 7

Mix of diols **4** and **5** (300 mg, 1.74 mmol), butanediol (BD) (366 mg, 4.06 mmol), dimethyl succinate (DMS) (651 mg, 4.06 mmol), together with Titanium(IV) butoxide (TBT) (4.75 mg, 0.039mmol) were added in a stirred glass reactor with a thermostatted salt bath. In the first stage, the temperature was raised to 180°C, keeping the system under N2 atmosphere for 3 h. In the second stage, the temperature was kept constant at 180°C, and the pressure was gradually reduced to 0.1 mbar. After 3 h, the reaction product was discharged from the reactor and cooled down to room temperature. A light yellow solid was obtained with 64% yield (570 mg, 2.85 mmol).

### Synthesis of Functionalized Copolyester 8

Copolyester **6** (201 mg, loading C=C 1.39 mmol/gr) was dissolved in 1 mL CH_3_CN in a quartz vial, and α-thioglycerol (121 μL, 1.40 mmol) was added. The vial was sealed with a septum, degassed by sparging for 5 min, and exposed to a handheld UV lamp (λ = 254 nm) for 7 h. The conversion was determined by ^1^H NMR integration of the terminal double bonds. The crude solution was then precipitated into cold CH_3_CN/H_2_O solution (1:1, 1.5 mL ^*^ 2) to yield the functional copolyester **8** (151 mg, 75%) as yellow solid.

### Synthesis of Functionalized Copolyester 9

Copolyester **6** (194 mg, loading C=C 1.70 mmol/gr) was dissolved in 1 mL CH_3_CN in a quartz vial, and 2-(Boc-amino)ethanethiol (192 mg, 1.08 mmol) and DMPA (4.6 mg, 0.02 mmol) were added. The vial was sealed with a septum, degassed by sparging for 5 min, and exposed to a handheld UV lamp (λ = 254 nm) for 23 h. The conversion was determined by ^1^H NMR integration of the terminal double bonds. The crude solution was then precipitated into cold CH_3_CN/H_2_O solution (1:1, 3 mL ^*^ 2) to yield the functional copolyester **9** (204 mg, 90%) as yellow solid.

## Results and Discussion

In this section, the results obtained were presented and discussed following this order: (1) optimization of the photooxidation of citronellol using zeolite supported-photosensitizer was reported with the aim to improve the yield and the regioselectivity of the process (section optimization of the regioselective photooxidation of citronellol); (2) secondly, the scalability and recyclability of the zeolite supported-photosensitizer (Thionine-NaY) were demonstrated [section scalability and recyclability of the zeolite supported-photosensitizer (Thionine-NaY)]; (3) the purified diol **4** and the unpurified mixture of diols **4**+**5** were employed for the preparation of two C-C double bond containing polyesters, which have been properly characterized (section synthesis and characterization of citronellol-containing biopolyesters **6** and **7**); (4) finally, we demonstrated the possibility to functionalize these C-C double bond containing polyesters using UV promoted thiol-ene click reaction (section synthesis of functionalized bio-polyesters **8** and **9** using UV-mediated thiol-ene reaction).

### Optimization of the Regioselective Photooxidation of Citronellol

We started comparing the photooxygenation reaction of (*rac*-)citronellol **1** with different sensitizers in homogeneous conditions and with zeolites (Table 1). As expected, complete conversion into a 1:1 mixture of regioisomers was obtained treating **1** with 1% RB, air and white light (entry 2), while only starting material was recovered in absence of the photosensitizer (entry 1). Similar results were obtained using methylene blue (MB) and thionine acetate (TH) in place of RB (entries 3 and 4). The experiments with zeolites were carried out after adsorption of the photosensitizers onto commercially available zeolite (NaY) followed by a cation-exchange process (Shailaja et al., 2000).

**Table 1:** Optimization of the regioselective photosensitized oxidation of citronellol **1** to give, after reduction, diols **4** and **5**.

**Entry**	**Sensitizer**	**Sens./zeolite [% w/w]**	**Solvent**	**1 [mg]**	**Time [h]**	**4: 5 ratio^**[a]**^**	**Conv. %^**[b]**^**	**Yield %^**[c]**^ 4 + 5**
1	–^[f]^	–	MeOH	500	8	–	0%	–
2^[d]^	RB^[g]^	–	MeOH	500	8	49:51	>95%	72%
3^[d]^	MB^[g]^	–	MeOH	500	7	48:52	>90%	63%
4^[e]^	TH^[g]^	–	MeOH	100	18	46:54	>95%	47%
5^[e]^	MB/NaY	0.001	Hexane	100	5.5	71:29	31%	–
6^[e]^	MB/NaY	0.001	Cyclohexane	220	19	70:30	71%	–
7^[e]^	MB/NaY^[h]^	0.001	Neat	20	7	81:19	98%	–
8^[e]^	MB/NaY^[h]^	0.001	Neat	100	29	82:18	51%	–
9^[e]^	MB/NaY	0.005	Neat	100	14	79:21	56%	–
10^[e]^	TH/NaY^[h]^	0.005	Neat	100	19	82:18	93%	87%
11^[e]^	TH/NaY^[h]^	0.005	Neat	50	15	82:18	92%%	66%
12^[e]^	TH/NaY^[h]^	0.005	Neat	50	23	88:12	100%	62%
13^[e]^	TH/NaY^[h]^	0.005	Neat	50	48	99:1	100%	41%
14^[e]^	TH/LiY^[h]^	0.005	Neat	50	15	99:1	92%	31%
15^[e]^	TH/NaY^[h]^	0.005	Neat	480	24	89:11	88%	78%

[a] The regioisomer ratio is calculated on the crude by ^1^H-NMR spectrum;

[b] Conversion, defined as [**4** + **5**] / starting **1**, is calculated on the crude by ^1^H-NMR;

[c] Isolated yield after column chromatography;

[d] NaBH_4_ (1.3 equiv) is used as reduction agent;

[e] PPh_3_ (1.5 equiv) is used as reducing agent;

[f] Reaction conditions: **1**, MeOH, air, white light, 8 h;

[g] Typical reaction conditions: **1** (1 equiv), photosensitizer (0.01 equiv), MeOH, air, white light, 8 h;

[h] *Typical reaction conditions: **1** (100 mg) in hexane (9 mL), zeolite-supported photosensitizer (1.8 gr), stirring 15 min in the dark; then evaporation of the solvent; the solid is treated with air, white light for 19 h; the hydroperoxides are recovered adding CH_3_CN and subsequent filtration*.

Since RB has never been immobilized on zeolites, we decided to study only the phenothiazine-based dyes (MB and TH), that, on the other hand, are known to be efficient sensitizers in photooxygenation reactions conducted in the presence of zeolites (Wahlen et al., 2004). According with the literature (Ramamurthy et al., 1993; Hoppe et al., 1995), we assume that in our samples the dye molecules are uniformly distributed and remain at the centers of the supercages of NaY. Actually, supercages of NaY are spherical and large enough to accommodate TH or MB (diameter of about 12 Å and a freevolume of 827 Å).

Firstly, we carried out the photooxidation reaction in a hexane slurry of MB-NaY and **1** (entry 5), finding good regioselectivity for compound **4**, though with low conversion, compared to the reaction conducted in standard conditions (entry 3). Although the zeolites showed improved selectivity, this experimental protocol clearly affected the efficiency of the reaction due to the multiphasic nature of the system. Actually, four different entities must interact in order to achieve high selectivity and conversion rate: the solid phase, containing the supported photosensitizer, the liquid phase, containing the solvent and the substrate, the gas phase, containing oxygen, and finally the light.

When we tried to prolong the irradiation time, we serendipitously discovered that, upon unexpected evaporation of the solvent, the reaction proceeded overnight under neat conditions, with improved conversion and regioselectivity (entry 6). Actually, in the absence of solvent, the MB-supported zeolite provides a microporous environment in which **1** may be adsorbed so to restrict its mobility, and may react with other local species, but is otherwise precluded from undergoing undesired further reactions (Rhodes, 2010).

Based on these results, we studied the photooxygenation in the absence of solvent, after adsorption of **1** onto the photosensitizer/zeolite system, varying the nature of the photosensitizer and the zeolite, the loading of the photosensitizer on zeolite and the loading of substrate respect on photosensitizer-supported zeolite. Following the typical loading levels used in literature (Shailaja et al., 2000; Pace and Clennan, 2002) (0.9 g of 0.001 % MB-NaY), 20 mg of **1** was successfully converted with a remarkable 81:19 regioisomeric ratio (entry 7), while the conversion rapidly dropped when the amount of substrate was increased (entry 8).

Thus, we considered the possibility to use different loading levels, preparing both 0.005% MB-NaY and TH-NaY. The latter showed superior performance, leading to the products in excellent isolated yield and with very high regioisomeric ratio (entries 9–10). Thus, we choose TH-NaY with 0.005% loading level as the best photosensitizer system. Further experiments demonstrated that the irradiation time affects the yield, possibly due to the selective decomposition of the tertiary hydroperoxide **3** (see entries 11–13). When 0.005% TH-LiY was used, extensive degradation appeared, as showed by low isolated yield (entry 14) (Kaanumalle et al., 2002). Although the more benign NaBH_4_ can be used for reducing hydroperoxides into alcohols, PPh_3_ allowed us to avoid a liquid-liquid extraction and thus the use of large amount of organic solvent.

### Scalability and Recyclability of the Zeolite Supported-Photosensitizer (Thionine-NaY)

**Figure 1:**
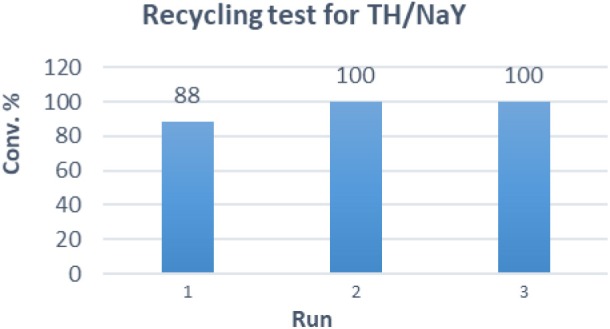
Recycling tests of TH/NaY (loading 0.005% w/w). Reaction conditions: **1** (480 mg), TH/NaY (8.7 gr), air, white light, 24 h; then CH_3_CN, supported-PPh_3_ (1.5 eq.), RT, 3 days.

Finally, the scalability of our optimized procedure was evaluated, converting almost half gram of substrate (entry 15) with conversion, selectivity and isolated yield that seemed not to be affected by the scaling up (comparison with entry 10). The recyclability and stability of the TH/NaY system was investigated by reusing the catalyst in consecutive catalytic runs (Figure 1). The results showed that substrate **1** can be nearly completely converted after 24 h of irradiation for at least three runs. After the run, TH/NaY was simply recovered by filtration, washed with CH_3_CN, and dried overnight at 110°C. This protocol was found to be crucial, as the photosensitizer/zeolite system is very sensitive to humidity and a significant decrease of activity is generally observed when it is not properly dried.

### Synthesis and Characterization of Citronellol-Containing Biopolyesters 6 and 7

**Scheme 2:**
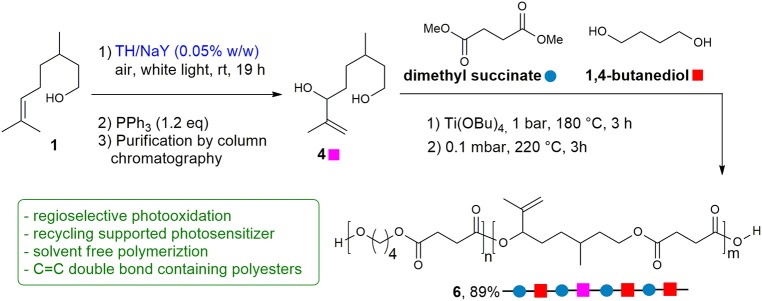
Photosensitized transformation of citronellol **1** into diol **4**, and polymerization process of functionalized co-polyester **6**. Reaction conditions: (1) neat, Ti(OBu)_4_ (150 ppm of Ti/g of polymer), 1 bar, 180°C, 3 h; (2) 0.1 mbar, 220°C, 3 h.

To demonstrate whether the purified diol **4** could be used for polymerization, a random copolyester was synthesized by two-stage polymerization, as summarized in Scheme 2, starting from: dimethyl succinate (DMS), butanediol (BD) and **4**. The reaction was carried out in bulk, starting from a BD/**4** molar ratio of 60/40. The glycols were added with a 30 mol% excess with respect to DMS. The reaction was conducted in a stirred glass reactor with a thermostated salt bath. Temperature and torque were continuously recorded during the polymerization process. In the first stage, the temperature was raised to 180°C, keeping the system under N_2_ atmosphere for 3 h. In the second stage, the temperature was slowly increased to 220°C, and the pressure was gradually reduced to 0.1 mbar. After 3 h, the reaction product was discharged from the reactor and cooled down to room temperature. Applying these conditions, copolymer **6** was successfully obtained in 89% yield as a light yellow solid.

Through the optimization of the polymerization reaction conditions (time, temperature and catalyst amount) it was possible to overcome the problem relative to the lower reactivity of the secondary hydroxyl group of diol **4**, avoiding at the same time crosslinking reactions involving the carbon double bond, which remains unchanged in the copolymer for subsequent ad hoc functionalization.

**Scheme 3:**
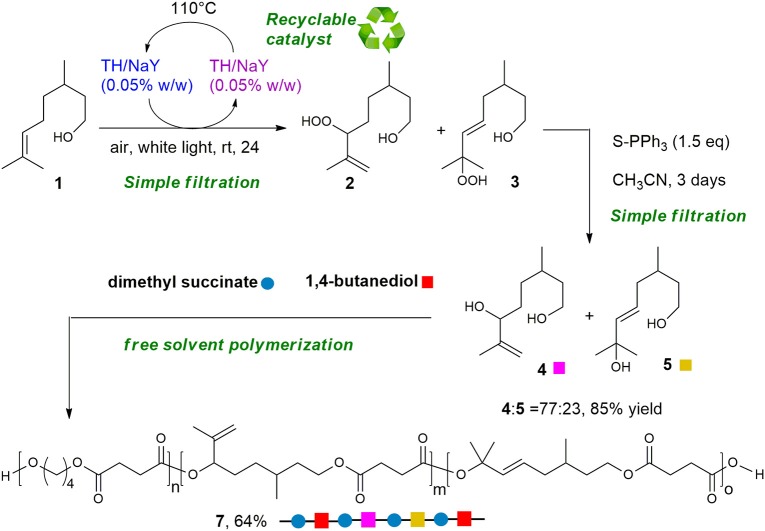
Green synthesis of functionalized-copolyester **7** starting from citronellol **1**

Encouraged by the above results, we decided to perform the polymerization process with the unpurified mixture of diols **4** and **5**, directly obtained through the regioselective-photosensitized oxidation and reduction process. The successful polymerization of the regioisomeric mixture, in fact, could avoid the chromatographic purification of **4**, that represents a drawback in the development of a sustainable procedure. Nevertheless, the polymerization of the mixture is in principle particularly challenging as diol **5**, beside the carbon double bond alike diol **4**, contains an even less reactive tertiary hydroxyl group. First, polymerization was carried out using previous conditions, resulting not successful. On increasing BD/**4** molar ratio to 70/30 and keeping constant the temperature at 180°C, a light yellow solid was obtained in 64% yield (Scheme 3).

It must be underlined that the proposed strategy represents a green method to transform a biobased substrate as citronellol into useful diols readily employed in the synthesis of a functionalized copolyester. Actually, the diols can be isolated after two simple filtrations, the first to recover **4** and **5** from the supported catalyst, the second to remove the supported reducing agent. Moreover, the catalyst can be conveniently reused after being dried, and the polymerization has been performed in bulk without solvents.

The thermal stability of the copolyesters were tested by thermogravimetric analysis (TGA). The thermogravimetric curves acquired under nitrogen flow (see Supplementary Material) shows a one-step weight loss with high temperature of maximum degradation rate (T_max_) (Table 2). The results show that the introduction of both **4** and **5** in the PBS main polymer chain does not compromise the good thermal stability of the neat homopolymer, being the T_max_ of both copolymers even higher than T_max_ of PBS.

**Table 2:** Molecular, structural, thermal and mechanical characterization data of PBS and copolymers **6**, **7** and **8**.

		**PBS**	**6**	**7**	**8**
^1^H-NMR	co-unit mol%	–	35	20 + 5	27
	Yield [%]	96	89	63	75
Viscosity	I.V. [dl/g]	1.48	0.79	1.06	0.67
TGA	T_max_ [°C]	399	409	404	259, 395
DSC	T_m_ [°C]	115	53	85	51
I SCAN	ΔH_m_ [J/g]	81	30	45	15
DSC	T_g_ [°C]	−34	−33	−34	−16
II SCAN	ΔCp [J/g*°C]	0.212	0.481	0.223	0.573
	T_c_ [°C]	/	/	23	/
	ΔH_c_ [J/g]	/	/	12	/
	T_m_ [°C]	115	/	85	/
	ΔH_m_ [J/g]	62	/	40	/
WAXS	*X*_c_ [%]	40	16	27	11
Tensile Test	σ_b_ [MPa]	337 ± 27	2.3 ± 0.8	7 ± 1	n.d
	ε_b_ [%]	4 ± 1	15 ± 2	5 ± 1	n.d
	E [MPa]	301 ± 2	26 ± 5	147 ± 16	n.d

The differential scanning calorimetry (DSC) heating curves (first and second scans after melt quenching) of the polymers under investigation (SI), with the relative calorimetric data (Table 2), show typical behavior of semicrystalline materials, being characterized by the glass-to-rubber transition step in heat capacity at low temperature, followed by an endothermic peak at higher temperature ascribable to the melting of the crystalline phase. A clear effect of copolymerization on both the position and the area of the melting peak can be evidenced. In particular, the introduction of the **4** and **5** subunits determines a decrease of melting temperature (T_m_), and of the associated enthalpy (ΔH_m_). This reduction seems to depend only on the subunit amount and not on their nature. As a matter of fact, the T_m_ decrease is higher in **6**, containing 35 mol% of **4**, than in **7**, in which two different co-units (20 and 5 mol% of **4** and **5**, respectively) have been introduced. This result can be considered as indirect evidence that the crystalline phase present in the copolymers is the same as the pure PBS, and the comonomeric units are completely rejected in the amorphous regions.

The amount of co-units also plays a role in the crystallization capability, as confirmed by the second scan traces. In fact, as one can see from Table 2, the high crystallization capability of PBS polymer chains is reduced in **7** containing 25 mol% of diols and is completely depressed in **6** by adding 35 mol% of **4**. Copolyester **7**, indeed, shows a more intense glass transition step with respect to PBS homopolymer, indicating higher amount of amorphous phase. The sample is able to crystallize during the heating scan as evidenced by the exothermic peak at 23°C (“cold-crystallization”). Nevertheless, being ΔH_m_ > ΔH_c_, the crystallization of **7** also partially occurs during the cooling step. On the other hand, the DSC second scan curve of **6**, evidenced that the introduction of 35 mol% of diol **4** allows to suppress crystallization, obtaining a completely amorphous material upon quenching, preventing the formation of crystal during the subsequent heating step. In fact, only the glass transition phenomenon can be detected.

Moreover, as suggested by the constant T_g_ values in all the three samples, the presence of **4** and **5** along the polymer backbone does not change the chain mobility.

The copolyesters films were also subjected to WAXS characterization to determine the crystal lattice. The X-ray patterns (SI) and the relative crystallinity degree (X_c_) are reported (Table 2). In agreement with the calorimetric results, the crystalline phase found in both the copolyesters corresponds to the α-PBS lattice with the characteristic peaks located at 19.6°, 22.5°, 21.7°, 25° and 45° (see Supplementary Material). Moreover, diffractometric peak position does not change with copolymerization, suggesting the rejection of the comonomeric units in the amorphous phase. The diffractometric results are in line with the calorimetric ones, also in relation to a reduction of the quantity of crystalline domains. Again, the higher the amount of co-units, the lower the crystallinity degree.

The polymer films obtained by compression molding were subjected to stress-strain measurements to evaluate their mechanical response (Table 2 and Supplementary Material). The results obtained evidenced a reduction of elastic modulus (E) and tensile strength (σ_b_) in both copolymers. In particular, with respect to PBS, E is halved in **7** and is five times lower for **6**.

The trend observed can be explained on the basis of the crystallinity decrease. As well known, both E and σ_b_ are directly dependent on the amorphous/crystals phase ratio, in particular a reduction of ordered regions determines the decrease of the elastic modulus and tensile strength. As concerns, the elongation at break (ε_b_), there is an increase from 4% for PBS film to 22% in the less crystalline copolyester **6**, while no significant effect was evidenced for **7**, which shows the same elongation at break of the homopolymer. Again, the dissimilar behavior can be related to the different degree of crystallinity, being this quantity higher for **7**.

### Synthesis of Functionalized Bio-Polyesters 8 and 9 Using UV-Mediated Thiol-Ene Reaction

**Scheme 4:**
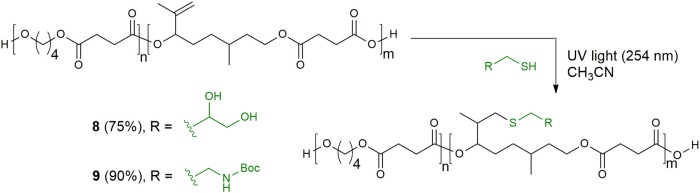
Thiol–ene modification of the citronellol-based copolyester of poly(butylene succinate) with thioglycerol and *N*-Boc-cysteamine.

Finally, we have investigated the possibility to functionalize the unsaturated copolyester **6** exploiting the highly efficient thiol–ene coupling reaction. The great success of this reaction in polymer and material science is due to its orthogonality to a wide range of functional groups and to the robustness of the ligation motif between substrates thanks to the stability of the thio-ether linkage in a wide range of chemical environments (Lowe, 2010). Thus, we carried out the reaction between polymer **6** and thioglycerol, using UV irradiation and acetonitrile, monitoring the conversion of the substrate by ^1^H-NMR analysis. After 6 h the double bond was quantitatively converted and the functionalized copolyester **8** was recovered by precipitation in good yield. When *N*-Boc-cysteamine was employed as thiol component, the addition of a small amount of photoinitiator 2,2-dimethoxyphenylacetophenone (DMPA, 0.05 eq) showed better results (Scheme 4).

While some examples of post-functionalized polyesters obtained combining ring-opening polymerization (ROP) and thiol–ene click chemistry recently appeared in literature (Darcos et al., 2012; Pelegri-O'Day et al., 2017; Guindani et al., 2019), to the best of our knowledge, this is one of the rare examples of combination of polycondensation reaction and thiol–ene click chemistry to prepare functionalized biobased degradable polyesters, and the first example employing isopropenyl moiety as reactive alkene substrate (Kolb and Meier, 2013; Trotta et al., 2019).

The successful incorporation of the two thiols into the polymer chains of citronellol-based copolyester was verified by IFT-IR measurements of the polymer films after purification. The ATR-FT-IR spectra of the functionalized polymers are shown in the Supporting Information (Figure S9) and compared to PBS homopolymer and copolyesters **6** and **7**. The inclusion of thioglycerol in polymer **8** can be deduced by the appearance of broad IR absorption band peaked around 3,450 cm^−1^, attributed to the stretching vibration of O-H groups. On the other hand, the functionalization with *N*-Boc-cysteamine results in two characteristic bands related to N-H stretching (around 3,370 cm^−1^) and bending (1,510 cm^−1^, associated to NH bending *trans* to carbonyl oxygen).

The functionalized copolyester **8** has been subjected to further characterization. As one can see from the data reported in Table 2, the functionalization determines a 15% reduction of intrinsic viscosity (proportional to the polymer molar mass) that however keeps quite high. As concerns the thermal stability, a two-step degradation can be evidenced: the first weight loss takes place at 259°C (related to the degradation of the co-units) and the second one at 395°C (ascribable to the degradation of BS sequences).

Calorimetric measurements highlight the introduction of -S-R branches provides a further reduction of the crystalline portion (ΔH_m_ decreases) with respect to the amorphous one (ΔCp increases). In addition, -S-R branches determines a reduction of chain mobility as indicated by the rise in T_g_.

## Conclusions

In the present work, we have successfully applied the dye-sensitized photooxidation reaction as sustainable route for the regioselective conversion of a naturally occurring monoterpenoid, citronellol, into diols **4** and **5**. The two-step sequence involves a photooxidation reaction performed in neat conditions using air, visible light and zeolite supported-Thionine as unique reagents, and secondly a reduction step mediated by a phosphine, to give **4** with high regioselectivity. The zeolite supported photosensitizer can be recovered and reused as catalyst for at least 3 times. Both the purified diol **4**, and the mixture of **4** and **5**, have been successfully used in a polycondensation reaction generating two copolyesters of poly(butylene succinate) (PBS). The introduction of the citronellol-based building block produced a more flexible and functionalizable material, providing an efficient way to modulate the final polymer properties, such as hydrophilicity, crystallinity, elasticity, and, eventually, bioactivity. The presence of the C=C double bond on the polymer has been exploited for the subsequent functionalizations, using a UV promoted thiol-ene click reaction. Two different functionalized copolyesters were synthetized and partially characterized. The application of these materials for controlled-drug delivery systems is underway in our laboratory and will be presented in due course.

## Data Availability Statement

All datasets generated for this study are included in the article/Supplementary Material.

## Author Contributions

The manuscript was written through contributions of all authors. All authors have given approval to the final version of the manuscript.

### Conflict of Interest

The authors declare that the research was conducted in the absence of any commercial or financial relationships that could be construed as a potential conflict of interest.

## Supplementary Material

Photographs of reactors and lamp used for photosensitized oxidation of citronellol, preparation of LiY zeolites from NaY, structure and composition of copolyesters **6**, **7** and **8** verified via ^1^H-NMR spectroscopy, thermogravimetric curves of copolyesters **6** and **7**, DSC heating curves of copolyesters **6** and **7**, WAXS characterization of copolymers **6** and **7**, evaluation of the mechanical properties of copolymers **6** and **7**, ^1^H and ^13^C NMR spectra of compound **4** and **5**, ATR-FTIR spectra of PBS and copolyesters **6**, **7**, **8** and **9**.
