# Supplementary material for: Regioselective Photooxidation of Citronellol: A Way to Monomers for Functionalized Bio-Polyesters
Source: Front Chem. 2020 Feb 13;8:85. doi: 10.3389/fchem.2020.00085 (PMC7031484; doi:10.3389/fchem.2020.00085)
Supplement: Supplementary file 1 [file Table_1.DOCX]

Supplementary Material

# Table of contents

Reactors used for photosensitized oxidation of citronellol 2

White Lamp emission spectrum 2

Photoreactor setup 3

Preparation of LiY zeolites from NaY 3

Structure and composition of copolyesters **6**, **7** and **8** verified *via* ^1^H-NMR spectroscopy 4

Thermogravimetric curves of copolyesters **6** and **7**  6

DSC heating curves of copolyesters **6** and **7** 7

WAXS characterization of copolymers **6** and **7** 8

Evaluation of the mechanical properties of copolymers **6** and **7** 9

^1^H and ^13^C NMR spectra of compound **4** and **5** 10

ATR-FTIR spectra of PBS and copolyesters **6**, **7**, **8** and **9** 12

References 12

# Reactors used for photosensitized oxidation of citronellol
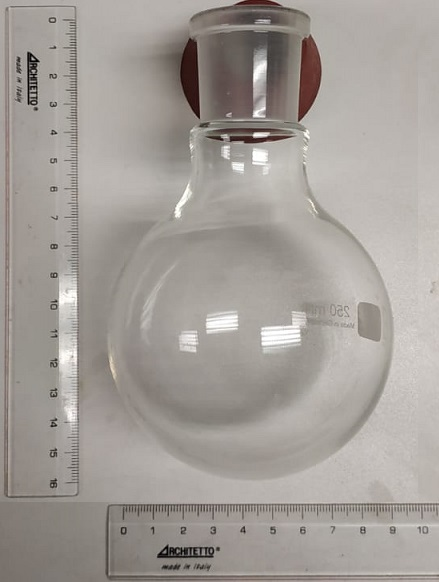

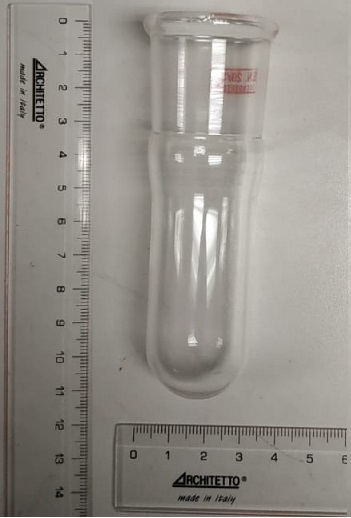

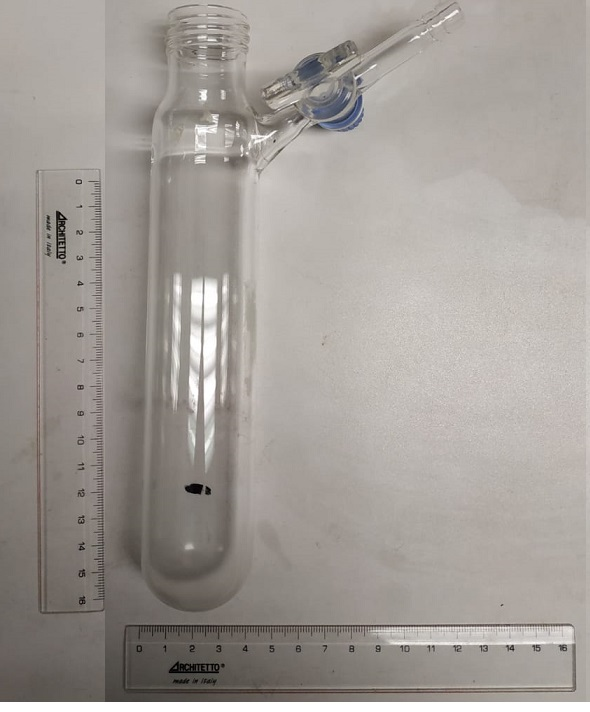


**c**

**b**

**a**

**Figure S1** Reactor **a** is used for the photooxidation in solution; reactor **b** is used for the photooxidation using Thionine/NaY photosensitizer (100 mg scale); reactor **c** is used for the photooxidation using Thionine/NaY photosensitizer (500 mg scale) and for the recycling tests.

# White Lamp emission spectrum

The emission spectrum of the white lamp used for the photosensitized oxidation reaction has been collected applying the optical fiber Ocean Optics and the AvaSpec-ULS4096CL-EVO (CMOS). The measurements are carried out on the lamp directly (blue line), and in the presence of pyrex reactor (orange reactor).

**
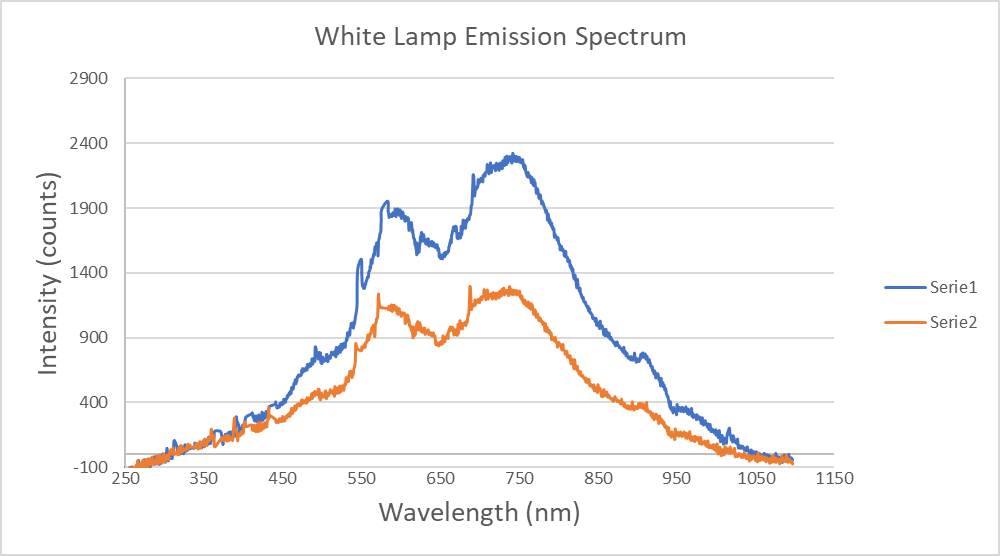
**

**Figure S2** Emission spectrum of the white lamp used for the photosensitized oxidation reaction

# Photoreactor setup to carry out the photooxidation in homogeneous (left) and heterogeneous (right) conditions

Photoreactor setup A is formed by a schlenk tube (reactor **a**, Figure S1) where the mixture of substrate, photosensitizer and solvent were posed (Figure S3, left). Air was dosed into the reaction using a Behringer workstation with pressure regulator HPP003SV.

Photoreactor setup B is based around a rotary evaporator fitted with reactor **b** or **c** depending on the scale of the reaction (Figure S3, right). Air was dosed into the reaction using a Behringer workstation with pressure regulator HPP003SV.

**
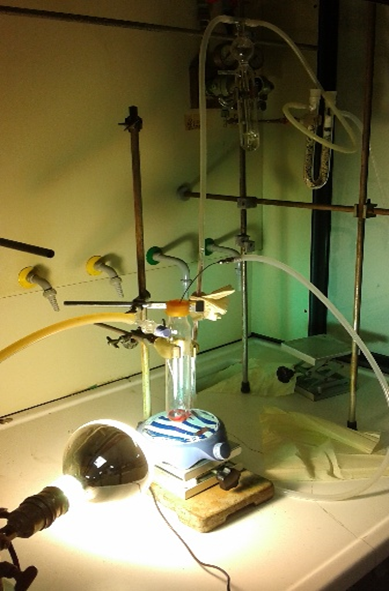

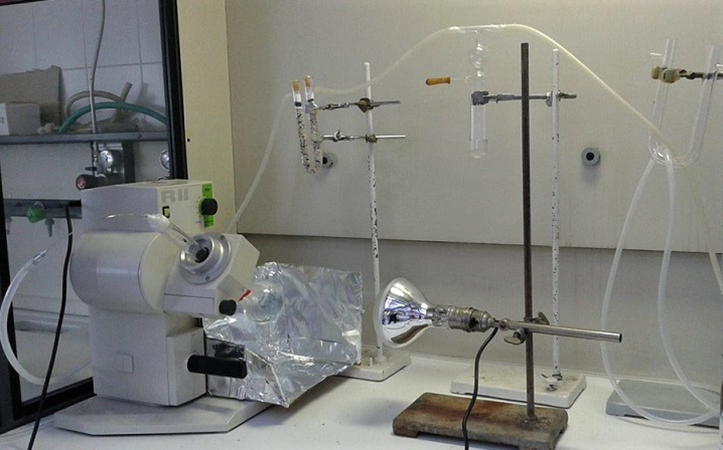
**

**Figure S3** Photoreactor setup A (left) and Photoreactor setup B (right)

# Preparation of LiY zeolites from NaY

Zeolite Y, lithium was prepared following a literature procedure ([Shailaja et al., 2000](#_ENREF_1)): a mixture of NaY zeolite (10 gr) in an aqueous solution of LiNO_3_ (250 mL, 10% w/vol) is mixed through an orbital shaker for 24 h at room temperature. Then the mixture is filtered, washed with deionized water and dried. The above procedure is repeated three times to ensure maximum exchange of cations. Finally, the zeolite is dried overnight at 110 °C.

# Structure and composition of copolyesters 6, 7 and 8 verified via 1H-NMR spectroscopy

^1^H-NMR analyses of copolyesters **6** and **7** confirm the exclusion of the occurrence of unwanted side reactions (crosslinking reactions) during the polymerization process. In particular, the double bond peak *6* at 4.90 ppm is still present in the copolymers chain.

The amount of subunit **4** in copolymer **6** has been determined from the ratio between the normalized area of the signal *10* located at 0.90 ppm ascribed to the methyl hydrogen atoms (3H) of 4 and the normalized area of the peak at 4.10 ppm corresponding to the methylene protons *1* (4H) and *12* (2H) of both butanediol and 4 subunits.

The amount of **4** and **5** in copolymer **7**, has been calculated from the ratio between the normalized are of signal *10* and *16* (3H), at 0.90 ppm, ascribed to the methyl groups of **4** and **5** and the normalized signal *1* (4H), *12* (2H) and *19* (2H) at 4.10 ppm, corresponding to the -CH_2_- protons in α position to carboxylic group for all the three glycolic sub-units. The percentage of **4** with respect to **5**, was calculated from the ratio between the intensity of peak *4* (1H) at 5.10 ppm and the normalized area of signals *10* (3H) and *16* (3H).

The amount of thioglycerol unit in copolymer **8**, has been calculated from the ratio between the normalized are of signal *5* (3H), at 1.00 ppm, ascribed to the methyl groups near to the thiol-ether moiety and the normalized signal *1* (4H) and *12* (2H) at 4.10 ppm, corresponding to the -CH_2_- protons in α position to carboxylic group for all the three glycolic sub-units.


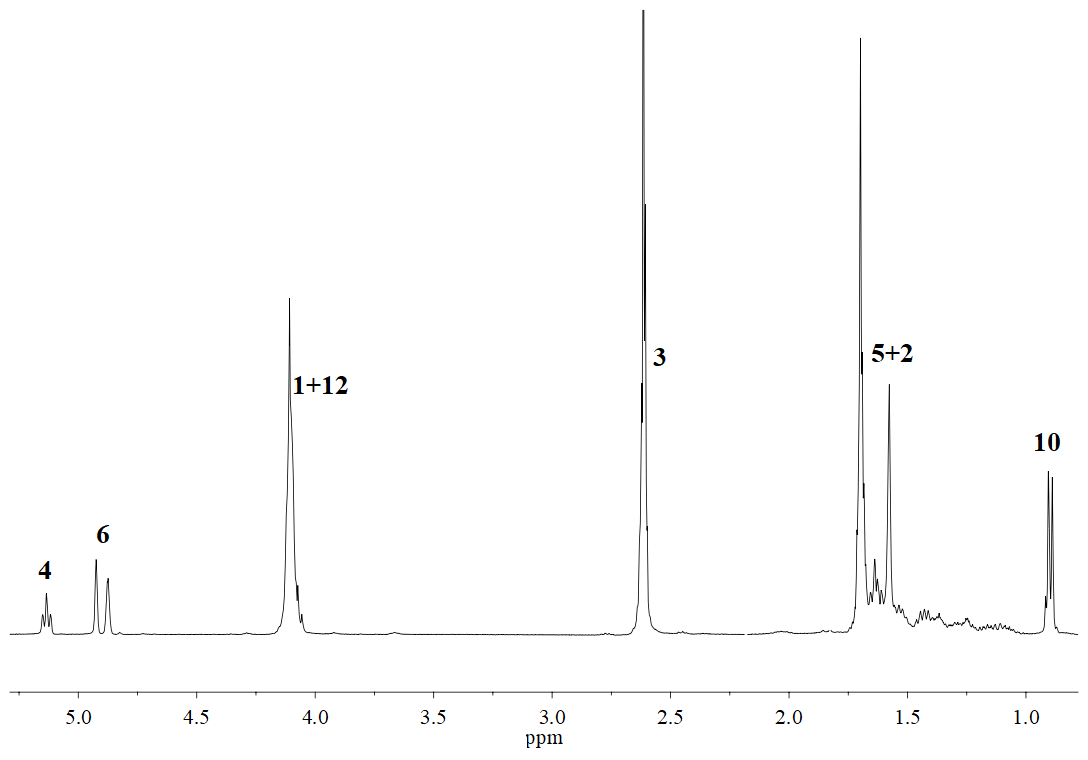


**7,8,9,11**

**Copolymer 6**


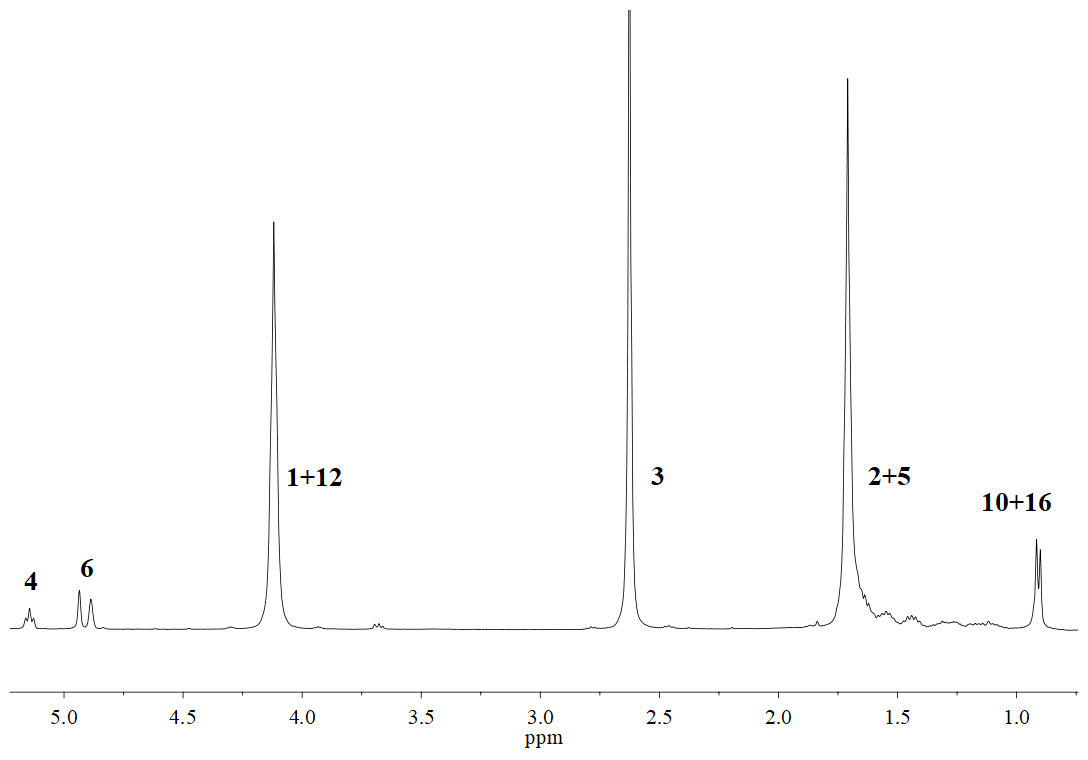


**7,8,9,11,13,14,15**

**+19**

**Copolymer 7**

**+19**


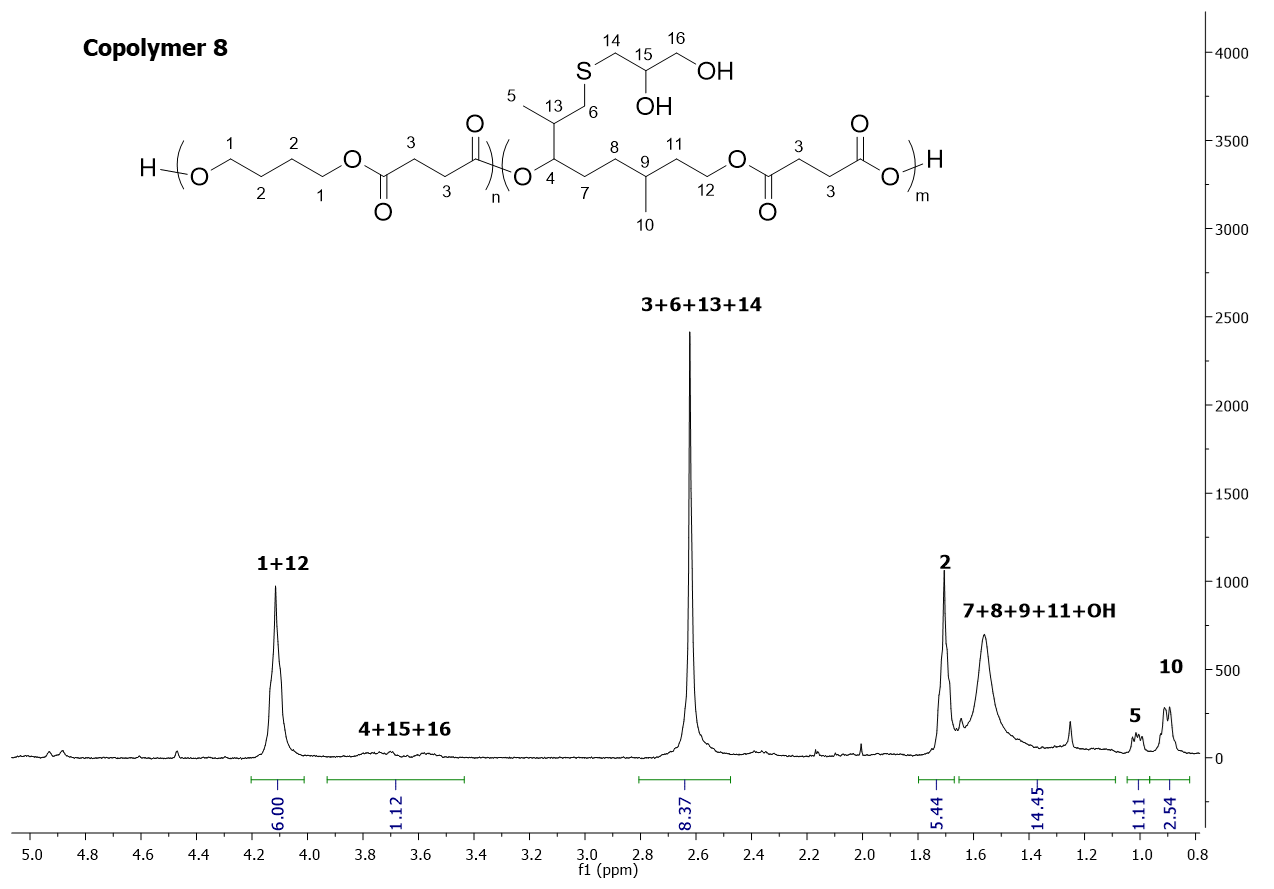


**Copolymer 8**

**Figure S4** ^1^H-NMR spectra of **6**, **7** and **8** with the corresponding molecular structure and peak assignment.

# Thermogravimetric curves acquired under nitrogen flow and the corresponding temperature of maximum degradation rate (Tmax)

**Figure S5** TGA curves of PBS (black), copolymer **6** (green) copolymer **7** (blue) acquired by heating at 10°C/min under N_2_ gas flow (40 ml/min).

# DSC heating curves (first (a) and second scan after melt quenching (b))

#

**b)**

**a)**

**Figure S6** DSC curves of PBS (black), copolymer **6** (green) copolymer **7** (blue): first (a) and second scan (b) after melt quenching. Heating rate 20°C/min.

# WAXS characterization of copolymers 6 and 7

**Figure S 7** WAXS patterns of PBS (black), copolymer **6** (green) copolymer **7** (blue).

# Evaluation of the mechanical properties of copolymers 6 and 7

F**igure S8** Stress-strain curves of PBS (black), copolymer **6** (green) copolymer **7** (blue).

# ^1^H and ^13^C NMR spectra of compound 4 and 5

# ATR-FTIR spectra of PBS and copolyesters 6, 7, 8 and 9

**

**

**Figure S9** ATR-FTIR spectra of the indicated polymer films in different spectral regions**.**

# Reference:

Shailaja, J., Sivaguru, J., Robbins, R.J., Ramamurthy, V., Sunoj, R.B., and Chandrasekhar, J. (2000). Singlet Oxygen Mediated Oxidation of Olefins within Zeolites: Selectivity and Complexities. *Tetrahedron* 56(36)**,** 6927-6943. doi: https://doi.org/10.1016/S0040-4020(00)00513-5.
